# Association between a lifestyle score and all-cause mortality: a prospective analysis of the Chilean National Health Survey 2009–2010

**DOI:** 10.1017/S1368980023002598

**Published:** 2023-12-06

**Authors:** Fanny Petermann-Rocha, Felipe Diaz-Toro, Claudia Troncoso-Pantoja, María Adela Martínez-Sanguinetti, Ana María Leiva-Ordoñez, Gabriela Nazar, Yeny Concha-Cisternas, Ximena Díaz Martínez, Fabian Lanuza, Fernanda Carrasco-Marín, Miquel Martorell, Karina Ramírez-Alarcón, Ana María Labraña, Solange Parra-Soto, Marcelo Villagran, Nicole Lasserre-Laso, Igor Cigarroa, Lorena Mardones, Jaime Vásquez-Gómez, Carlos A Celis-Morales

**Affiliations:** 1 Centro de Investigación Biomédica, Facultad de Medicina, Universidad Diego Portales, Santiago, Chile; 2 Department of Epidemiology, Mailman School of Public Health, Columbia University, New York, NY, USA; 3 Facultad de Enfermería, Universidad Andres Bello, Santiago 7550196, Chile; 4 Centro de Investigación en Educación y Desarrollo (CIEDE-UCSC), Departamento de Salud Pública, Facultad de Medicina, Universidad Católica de la Santísima Concepción, Concepción, Chile; 5 Instituto de Farmacia, Facultad de Ciencias, Universidad Austral de Chile, Valdivia, Chile; 6 Instituto de Anatomía, Histología y Patología, Facultad de Medicina, Universidad Austral de Chile, Valdivia, Chile; 7 Departamento de Psicología, Facultad de Ciencias Sociales, Universidad de Concepción, Concepción, Chile; 8 Centro de Vida Saludable, Universidad de Concepción, Concepción, Chile; 9 Escuela de Kinesiología, Facultad de Salud, Universidad Santo Tomás, Talca, Chile; 10 Pedagogía en Educación Física, Facultad de Educación, Universidad Autónoma de Chile, Talca, Chile; 11 Departamento de Ciencias de la Educación, Grupo calidad de Vida en diferentes Poblaciones, Universidad del Biobio, Chillán, Chile; 12 Departamento de Procesos Diagnósticos y Evaluación, Facultad de Ciencias de la Salud, Universidad Católica de Temuco, Temuco 4813302, Chile; 13 School of Cardiovascular and Metabolic Health, University of Glasgow, Glasgow, UK; 14 Departamento de Enfermería, Farmacología y Fisioterapia, Facultad de Medicina y Enfermería, Universidad de Córdoba, Córdoba, España; 15 Departamento de Nutrición y Dietética, Facultad de Farmacia, Universidad de Concepción, Concepción, Chile; 16 Departamento de Nutrición y Salud Pública, Facultad Ciencias de la Salud y de los Alimentos, Universidad del Bío-Bío, Chillán 3780000, Chile; 17 Laboratorio de Ciencias Biomédicas, Facultad de Medicina, Universidad Católica de la Santísima Concepción, Concepción, Chile; 18 Escuela de Nutrición y Dietética, Facultad de Salud, Universidad Santo Tomás, Los Ángeles, Chile; 19 Escuela de Kinesiología, Facultad de Salud, Universidad Santo Tomás, Los Ángeles 4440000, Chile; 20 Centro de Biodiversidad y Ambientes Sustentables (CIBAS) Universidad Católica de la Santísima Concepción, Concepción, Chile; 21 Centro de Investigación de Estudios Avanzados del Maule (CIEAM), Laboratorio de Rendimiento Humano, Universidad Católica del Maule, Talca, Chile; 22 Human Performance Lab, Education, Physical Activity and Health Research Unit, Universidad Católica del Maule, Talca, 3466706, Chile

**Keywords:** Lifestyle, prospective study, mortality

## Abstract

**Objective:**

To investigate the association between a lifestyle score and all-cause mortality in the Chilean population.

**Design:**

Prospective study.

**Settings:**

The score was based on seven modifiable behaviours: salt intake, fruit and vegetable intake, alcohol consumption, sleep duration, smoking, physical activity and sedentary behaviours. 1-point was assigned for each healthy recommendation. Points were summed to create an unweighted score from 0 (less healthy) to 7 (healthiest). According to their score, participants were then classified into: less healthy (0–2 points), moderately healthy (3–4 points) and the healthiest (5–7 points). Associations between the categories of lifestyle score and all-cause mortality were investigated using Cox proportional hazard models adjusted for confounders. Nonlinear associations were also investigated.

**Participants:**

2706 participants from the Chilean National Health Survey 2009–2010.

**Results:**

After a median follow-up of 10·9 years, 286 (10·6 %) participants died. In the maximally adjusted model, and compared with the healthiest participants, those less healthy had 2·55 (95 % CI 1·75, 3·71) times higher mortality risk due to any cause. Similar trends were identified for the moderately healthy group. Moreover, there was a significant trend towards increasing the mortality risk when increasing unhealthy behaviours (hazard ratio model 3: 1·61 (95 % CI 1·34, 1·94)). There was no evidence of nonlinearity between the lifestyle score and all-cause mortality.

**Conclusion:**

Individuals in the less healthy lifestyle category had higher mortality risk than the healthiest group. Therefore, public health strategies should be implemented to promote adherence to a healthy lifestyle across the Chilean population.

The WHO defines a healthy lifestyle as *‘a way of living that lowers the risk of being seriously ill or dying earlier’* ^(1)^. In this context, healthier lifestyle behaviours, such as performing physical activity, following a balanced diet and sleeping well, have been recognised for their protective role against non-communicable diseases and mortality^(2,3)^. Unfortunately, risk factors – those associated with changes in the risk of relevant conditions, health processes or outcomes^(4)^ – remain the principal accountable for the high burden of morbidity and mortality^(3)^. In 2020, the Global Burden of Disease identified that environmental pollution, the use of drugs, high blood glucose levels and high BMI were the main risk factors that increased between 1990 and 2019 worldwide^(3)^. For mortality, elevated systolic blood pressure and smoking were the principal causes accountable for deaths, attributing to 10·8 and 8·7 million deaths across the globe, respectively. In Chile, high blood pressure, high blood glucose levels and high body mass index (BMI) were identified as the main mortality risk factors^(3,5)^.

Although several studies have investigated the association of individual lifestyle factors with adverse health outcomes^(6–11)^, both protective and risk factors interact with each other and should not be investigated in isolation. For instance, people who sleep less are also more likely to have higher stress levels^(12,13)^. Consequently, studying only one health risk factor without considering others could mislead the true biological complexity association between lifestyle behaviours and mortality risk.

International studies have shown inverse associations between healthy lifestyle scores and all-cause mortality^(14–19)^. Even if there is no evidence from prospective cohort studies in Chile looking at the link between lifestyle behaviours and mortality risk, a few studies have looked at the association between single risk factors and health outcomes in the country^(7–9,20)^. Considering this gap, this study investigated the association between a lifestyle score and all-cause mortality in the Chilean population.

## Methods

This cohort study was based on participants aged ≥ 15 years from the Chilean National Health Survey 2009–2010 (CNHS 2009–2010). The CNHS 2009–2010 was a large, nationally representative population-based study of biological and lifestyle risk factors, dietary status and health, conducted every 6 years in Chile in both urban and rural zones. One participant was randomly selected per household, while pregnant individuals and individuals with violent behaviour excluded, as described elsewhere^(21)^. Data were collected by trained staff in two visits, in which individuals were administered questionnaires, and anthropometrical and physiological measures, as well as biological samples, were obtained. From the original sample size (5293 participants), 2706 participants had available data for the exposure and covariates and were, therefore, included in the analyses.

### Lifestyle score

Seven variables associated with lifestyle were used to create the lifestyle score as described elsewhere^(22)^: fruit and vegetable intake, salt intake, alcohol consumption, smoking, sleep duration, physical activity and sedentary behaviour. This score was created using as a reference a previous score created in an Australian cohort^(23)^ and updated for the UK Biobank study^(15)^ with the available variables in the CNHS 2009–2010^(21)^.

Dietary intake of fruit and vegetables was reported using a food frequency questionnaire (FFQ), which was administered once and was previously validated. Participants were asked, ‘In a typical/ordinary week, how many days do you eat fruit?’, ‘In a typical/ordinary week, how many days do you eat vegetables?’ and ‘How many servings of fruits/vegetables or vegetable salad do you eat in one of those days?’ which was then converted into gramme^(21)^. Salt intake was derived from a urine sample and estimated using a conversion formula derived by Tanaka *et al*.^(24)^. This formula was validated by the Chilean Ministry of Health and has been previously used elsewhere^(25)^. Alcohol consumption was self-reported and collected using the ‘Alcohol Use Disorders Identification Test’ (AUDIT) questionnaire developed by WHO^(26)^ and adopted for the Chilean population^(27)^. Smoking (non-smoker, ex-smoker or smoker) and sleep duration (in h/d) were self-reported using nationally validated questionnaires. Physical activity levels, including moderate and vigorous intensities and transport-related physical activity, were determined using the Global Physical Activity Questionnaire version 2 (GPAQ v2)^(28)^, previously validated^(29)^. Physical activity was then categorised into: inactive individuals (< 600 MET/min/week) and active individuals (≥ 600 MET/min/week)^(30,31)^. Sedentary behaviour was derived using the following question: ‘how much time do you usually spend sitting or reclining on a typical day?’^(30)^.

We assigned 1-point to participants for each following healthy recommendations they met, defined as: (i) ≥ 5 serving/d of fruit and vegetables; (ii) salt intake < 8 g/d (this classification was used since a previous document highlighted that the risk of hypertension increases over this consumption in this population)^(25)^; (iii) AUDIT score < 8 points; (iv) between 7–9 h/d of sleep^(32)^; (v) never smoking; (vi) being physically active (≥ 600 MET/min/week) and (vii) sitting time < 4 h/d^(33)^. If they did not meet the classification, 0-points were assigned at that category. Points from these seven lifestyle factors were summed to create an unweighted score ranging from 0 (people who did not meet any healthy recommendations) to 7 (people who met all seven recommendations). For this study, three categories were then created according to the score distribution: less healthy (scores between 0 and 2), moderately healthy (scores between 3 and 4) and the healthiest (scores between 5 and 7).

### All-cause mortality

The outcome of the current study was all-cause mortality. The date of death was obtained from death certificates linked to the Chilean Civil Registry and Identification. Mortality data were available until the 31st of December 2020. Therefore, mortality follow-up was censored on this date or the date of death if this occurred earlier.

### Covariates

Socio-demographic data were collected for all participants, including age, sex, zone of residence (rural or urban) and education level (primary (low), secondary (middle) or beyond secondary (high)), using nationally validated questionnaires^(21)^. Diabetes, hypertension and high cholesterol were self-reported using the following question: ‘Has a doctor, nurse, or another health professional ever told you that you have had or have: high cholesterol, diabetes or hypertension?’

BMI was calculated as weight/height^2^ and classified using the WHO criteria for adults (underweight: < 18·5 kg/m^2^; normal: 18·5–24·9 kg/m^2^; overweight: 25·0–29·9 kg/m^2^; obese: ≥ 30·0 kg/m^2^)^(34)^ and the Pan American Health Organization criteria for adults older than 60 years (underweight: < 23·0 kg/m^2^; normal: 23·0–27·9 kg/m^2^; overweight: 28·0–31·9 kg/m^2^; obese: ≥ 32·0 kg/m^2^)^(35)^.

### Statistical analyses

Descriptive characteristics are presented as means with standard deviation for continuous variables and as frequencies and percentages for categorical variables.

Associations between the categories of the lifestyle score and all-cause mortality were investigated using Cox proportional hazard models. Individuals in the healthiest category were used as the referent. The results are reported as hazard ratios (HR) and their 95 % confidence interval (CI). Duration of follow-up was used as the time-dependent variable.

Nonlinear associations between the continuous lifestyle score and all-cause mortality were investigated using penalised cubic splines fitted in Cox proportional hazard models. The penalised spline is a variation of the basis spline, which is less sensitive to knot numbers and placements than restricted cubic splines^(36)^. For the splines, values were truncated to less than 1 % and greater than 99 % of the score values. After truncation, the mean value of the score was used as a referent group.

Analyses were adjusted for confounding factors based on previous literature^(37,38)^, using the following three models: model 1 was adjusted for socio-demographic factors (age, sex, zone of residence and educational level). Model 2: as per model 1, but additionally included health-related factors (self-reported diabetes, hypertension and high cholesterol). Model 3: as per model 2, but additionally included BMI. In addition, one sensitivity analysis was conducted using a 2-year landmark, excluding all participants who died within the first 2 years of follow-up (*n* 39). This approach minimised the effect of reverse causality.

The rate advancement periods – which indicate the number of additional chronologic years that would be required to yield the equivalent risk rate for the healthiest *v*. less healthy individuals – were also estimated as described previously^(39,40)^. To calculate rate advancement periods, we divided the logarithm coefﬁcient (HR) for the mortality for the lifestyle categories referent to people in the healthiest category for the mortality associated with each yearly increase in age, for example, 
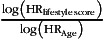
 .

To investigate whether the association between the lifestyle score categories and all-cause mortality differed by population groups, we tested for interactions and stratified the analyses by age (≥ and < 60 years), sex (men and women), zone of residence (urban and rural) and BMI categories (normal and overweight/ obese). Underweight participants were removed for the later stratification only.

Finally, associations between the seven components of the lifestyle score and all-cause mortality were investigated using Cox proportional hazard models. The normal range for each component was used as the reference group. For these analyses, two models were run: (i) model 3, using the same confounders mentioned above and (ii) model 4, as per model 3 and mutually adjusted for the individual components of the score when these were not the exposure.

Stata 18 and R 3.6.1 (using the packages ‘survival’ and ‘spline’) were used to perform the analyses. A *P*-value below 0·05 was considered statistically significant. This study followed the STROBE reporting guidelines for cohort studies^(41)^.

## Results

**Fig. 1:**
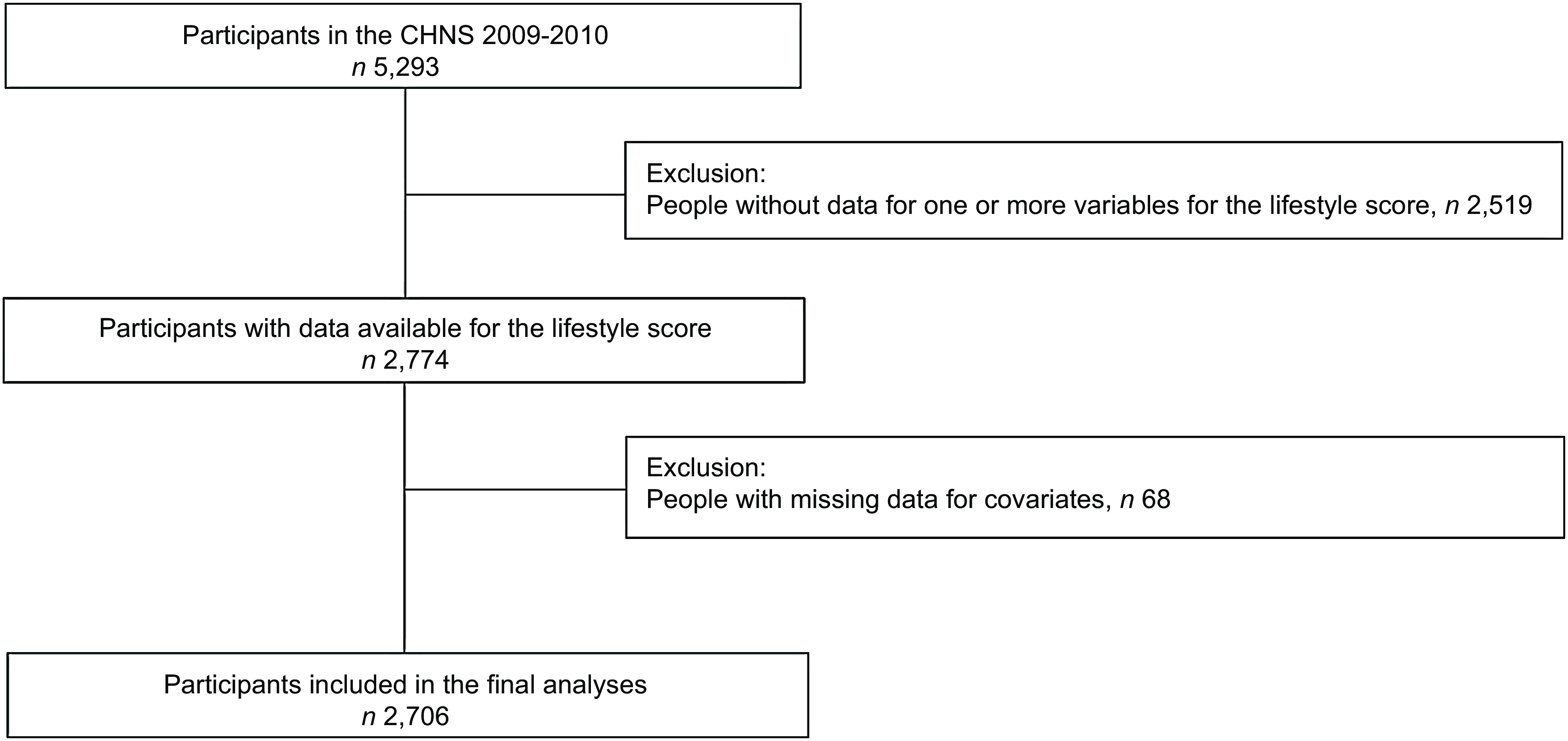
Participants included in the analyses

After excluding people with missing data for the lifestyle score and covariates, 2706 participants were included in the analyses (Fig. 1). Over a median follow-up of 10·9 years (interquartile range 10·6–11·0 years), 286 (10·6 %) participants died.

The baseline characteristics of the participants included by the lifestyle categories are shown in Table 1. Overall, the healthiest participants were more likely to be women with a lower BMI. In contrast, less healthy participants were more likely to live in urban areas, had lower educational levels and had a higher prevalence of hypertension and diabetes (Table 1).

**Table 1:** General characteristics of the study population by the lifestyle score categories at baseline

	Total		Healthiest		Moderately healthy		Less healthy	
	*n*	%	*n*	%	*n*	%	*n*	%
*n* (%)	2706	100	682	25·2	1581	58·4	443	16·4
Baseline age (years)								
Mean	46·7		46·5		46·2		46·2	
s d	18·4		17·5		18·5		19·4	
Sex								
Women	1582	58·5	451	66·1	925	58·5	206	46·5
Men	1124	41·5	231	33·9	656	41·5	237	53·5
Educational level								
Low < 8 years	530	19·6	124	18·2	304	19·2	102	23·0
Middle 8–12 years	1477	54·6	385	56·4	867	54·9	225	50·8
High > 12 years	699	25·8	173	25·4	410	25·9	116	26·2
Zone of residency								
Urban	2307	85·2	570	83·6	1349	85·3	388	87·5
Rural	399	14·8	112	16·4	232	14·7	55	12·4
Diabetes (yes)	219	8·1	46	6·7	122	7·7	51	11·5
Hypertension (yes)	865	32·0	211	30·9	510	32·3	144	32·5
High cholesterol (yes)	585	21·6	167	24·5	332	21·0	86	19·4
BMI (kg/m^2^)								
Mean	27·8		27·5		27·7		28·8	
sd	5·5		5·5		5·3		6·0	
BMI categories								
Underweight	140	5·2	44	6·4	80	5·0	16	3·6
Normal weight	892	33·0	248	36·4	521	33·0	123	27·8
Overweight	969	35·8	227	33·3	575	36·4	167	37·7
Obese	705	26·0	163	23·9	405	25·6	137	30·9

*N*, number.

**Fig. 2:**
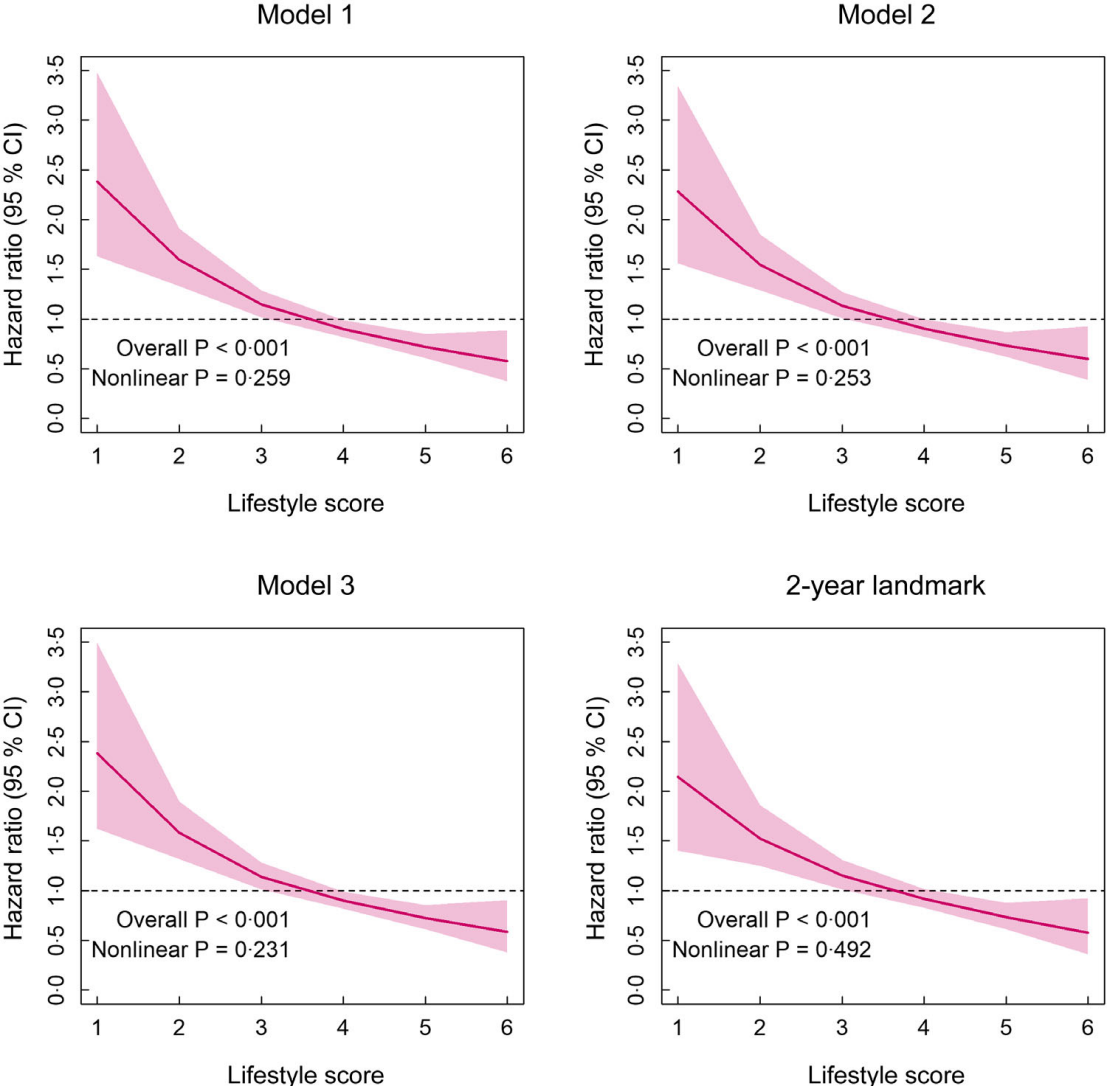
Associations between the continuous lifestyle score and all-cause mortality in Chilean adults. Analyses are presented as HR and their 95 % CI. All analyses were adjusted for age, sex, zone of residency, educational level, diabetes, hypertension, high cholesterol and BMI. 1-year and 2-year landmarks excluded people who died during the first and first 2 years of follow-up

Nonlinear associations between the lifestyle score and all-cause mortality are shown in Fig. 2. There was no evidence of nonlinearity between the lifestyle score and mortality in any model included (*P*-value > 0·05). A similar association was observed when a 2-year landmark was conducted.

Associations between the lifestyle score and all-cause mortality are presented in Table 2. In the minimally adjusted model, and compared with the healthiest participants, those less healthy had 2·62 (95 % CI 1·80, 3·79) times higher mortality risk due to any cause. After adjusting for health-related factors and BMI (models 2 and 3), the association previously observed was attenuated but remained statistically significant for those classified as less healthy (HR _model 3_:2·55 (95 % CI 1·75, 3·71)). Even if the associations were slightly lower, all models identified similar associations for the moderately healthy group (HR _model 3_ 1·50 (95 % CI 1·08; 2·10)). Moreover, there was a significant trend towards increasing the mortality risk when increasing unhealthy behaviours (HR _model 3_: 1·61 (95 % CI 1·34, 1·94)). Similar trends of associations were observed when the 2-year landmark analysis was included as a sensitivity analysis (Table 2).

**Table 2:** Associations between a lifestyle score and all-cause mortality in Chilean adults

		Healthiest	Moderately healthy			Less healthy			Trend		
All-cause mortality	Total cases/events	HR (95 % CI)	HR	95 % CI	*P*	HR	95 % CI	*P*	HR	95 % CI	*P*
Model 1	2706/ 286	1·00 (Ref.)	1·51	1·08, 2·10	0·015	2·62	1·80, 3·79	< 0·001	1·64	1·36, 1·97	< 0·001
Model 2	2706/ 286	1·00 (Ref.)	1·49	1·07, 2·07	0·019	2·46	1·69, 3·57	< 0·001	1·58	1·31, 1·90	< 0·001
Model 3	2706/ 286	1·00 (Ref.)	1·50	1·08, 2·10	0·016	2·55	1·75, 3·71	< 0·001	1·61	1·34, 1·94	< 0·001
2-year landmark*	2667/247	1·00 (Ref.)	1·47	1·04, 2·09	0·030	2·82	1·53, 3·41	< 0·001	1·52	1·24, 1·85	< 0·001

Analyses are presented as HR and their 95 % CI. Individuals in the healthiest category were used as the referent. Model 1: was adjusted for age, sex, zone of residency and educational level; model 2: as per model 1 but additionally for diabetes, hypertension and high cholesterol: model 3 as per model 2 but additionally for BMI. A 2-year landmark was carried out as a sensitivity analysis, excluding people who died during the first 2-year of follow-up.

* Using covariates from model 3.

When the analyses were stratified by age, sex, zone of residency and BMI categories (Table 3), similar trends of associations were identified. However, no significant interactions were observed for any subgroup (*P*_interaction_ > 0·05). Interestingly, compared with their very healthy counterparts, the strongest associations between the less healthy category and all-cause mortality were identified in individuals ≥ 60 years (HR: 2·74 (95 % CI 1·82, 4·14)), in women (HR: 3·45 (95 % CI 1·99, 6·00)), in urban areas (HR: 2·56 (95 % CI 1·69, 3·86)) and individuals with normal BMI (HR: 3·33 (95 % CI 1·70, 6·52)).

**Table 3:** Associations between lifestyle score and all-cause mortality by subgroups

		Healthiest	Moderately healthy			Less healthy		
All-cause mortality	Total cases/events	HR (95 % CI)	HR	95 % CI	*P*	HR	95 % CI	*P*
< 60 years	2000/ 56	1·00 (Ref.)	2·09	0·96, 4·51	0·062	2·70	1·08, 6·74	0·033
≥ 60 years	706 /230	1·00 (Ref.)	1·43	0·99, 2·06	0·059	2·74	1·82, 4·14	< 0·001
*P*_interaction_					0·471			0·761
Men	1124/ 147	1·00 (Ref.)	1·09	0·68, 1·74	0·724	1·85	1·11, 3·08	0·018
Women	1582/ 139	1·00 (Ref.)	2·01	1·25, 3·21	0·004	3·45	1·99, 6·00	< 0·001
*P*_interaction_					0·078			0·169
Urban	2307/238	1·00 (Ref.)	1·45	1·01, 2·08	0·046	2·56	1·69, 3·86	< 0·001
Rural	399/48	1·00 (Ref.)	2·08	0·89, 4·83	0·089	2·54	0·96, 6·72	0·061
*P*_interaction_					0·529			0·981
Normal	892/95	1·00 (Ref.)	1·95	1·09, 3·51	0·025	3·33	1·70, 6·52	< 0·001
Overweight/obese	1674/143	1·00 (Ref.)	1·39	0·86, 2·26	0·182	2·44	1·44, 4·13	0·001
*P*_interaction_					0·479			0·669

Analyses are presented as HR and their 95 % CI. Individuals in the very healthy category were used as the referent. All analyses were adjusted for age, sex, zone of residency, educational level, diabetes, hypertension, high cholesterol and BMI when these were not used as moderators. Underweight participants were removed just for the normal–overweight/obesity interaction analysis.

Based on rate advancement periods analyses, the mortality rate estimated for the healthiest participants was equivalent to those in the moderately healthy and unhealthy category but who were 4·7 (95 % CI 1·0, 7·78) and 10·9 (95 % CI 7·27, 13·8) years older, respectively.

Finally, of the seven components used to create the score, alcohol intake (HR: 1·68 (95 % CI 1·10, 2·56)), followed by sedentary behaviour (HR: 1·58 (95 % CI 1·24, 2·02)) and physical activity (HR: 1·51 (95 % CI 1·17, 1·94)) showed the highest mortality risk in the mutually adjusted model (see online supplementary material, Supplemental Table 1).

## Discussion

Using the CNHS 2009–2010, we identified that participants in the less healthy lifestyle category had the highest mortality risk due to any cause, independently of confounders. Moreover, our rate advancement periods analyses identified that people in the less healthy category might die up to 11 years earlier than their healthy counterparts. Our findings have important clinical implications as the population in Chile has experienced significant demographic changes, being one of the most aged of Latin American countries^(42)^. Therefore, considering the higher proportion of older individuals in the country^(43)^ and the higher prevalence of non-communicable diseases such as obesity, cardiovascular diseases, diabetes and hypertension^(21,44,45)^, public health strategies aiming to improve adherence to healthy lifestyle behaviours could contribute to reducing the excess risk mortality experienced by those with less healthy lifestyles.

Our primary findings were consistent with previous healthy lifestyle scores that positively associated a worse lifestyle with a higher risk of mortality in different regions across the globe^(14–19)^. For instance, Foster *et al*. – over a follow-up of 4·9 years of 328 594 UK Biobank participants – identified that those participants who followed a least healthy lifestyle had 2·06 times (95 % CI 1·86, 2·29) higher risk of dying compared with the healthiest participants^(15)^. Likewise, Lee *et al*. – using data from 37 472 Korean participants of the Korea National Health and Nutrition Examination Surveys (2007–2014) – highlighted that from five different lifestyle risk factors (smoking, alcohol, body weight, physical activity and sleep), individuals with four or five lifestyle risk factors had 2·01 time (95 % CI 1·43, 2·82) higher risk of all-cause mortality than those with zero lifestyle risk factors^(17)^. In the Netherlands, after a median follow-up of 12·4 years of 33 066 participants of the EPIC-NL study, those who adhered to the four healthy lifestyles investigated (smoking, BMI, physical activity and Mediterranean diet) lived 2 years longer than those who adhered to none^(14)^. Comparable results were reported in China by Wu *et al*.^(18)^, in Denmark by Ibsen *et al*.^(16)^ and in Spain by Ruiz-Estigarribia *et al*.^(19)^.

In South America, a subanalysis from the PURE study identified that the mains modifiable risk factors associated with a higher mortality risk were current use of tobacco, lower education levels (primary), former drinkers, low to moderate physical activity as well as hypertension, diabetes and abdominal obesity^(11)^. However, even if the results from the PURE study provide novel evidence regarding the landscape in South America, analyses were performed by individual risk factors and not as a cumulative score as we did in this study. In addition, the data provided summarise four countries, including Chile, but they did not present the individual associations by each of the countries included^(11)^. Therefore, our study provides a unique opportunity to fill gaps in the current literature, addressing our research question for the first time using Chilean data.

The inverse association between a less healthy lifestyle and all-cause mortality was stronger in women, older people and those living in urban areas. These results agree with those reported by Lee *et al*. in Korean adults, where women (HR: 3·30 (95 % CI 1·66, 6·57)) and people living in metropolitan cities (HR: 2·63 (95 % CI 1·58, 4·37)) with a worse lifestyle score had a higher risk of all-cause mortality^(17)^. However, in contrast to our study, the risk was higher in middle-aged adults (HR: 3·25 (95 % CI 1·55, 6·79)) than in older adults (HR: 1·50 (95 % CI 0·96, 2·35))^(17)^. The latter may be associated with the different cultural patterns in each country. On the other hand, in our study, less healthy individuals with normal BMI had a higher mortality risk than those less healthy but overweight or obese. This result is not surprising considering the previous evidence regarding non-obese but metabolically unhealthy individuals^(46,47)^, which emphasises the role of metabolic health in individuals regardless of their nutritional status. A previous Chilean study highlighted that 17·4 % of the population may be non-obese but metabolically unhealthy^(48)^.

### Strengths and limitations

The CNHS 2009–2010 is a nationally representative sample of the adult Chilean population. In addition, including a wide range of health, demographic and behavioural variables in the dataset allowed for a comprehensive adjustment of the effects of confounding factors. However, this study is not exempt from limitations. First, data were collected several years ago, and some lifestyle behaviours may likely have changed over time. However, it is the most up-to-date information available from Chile with linkage data. Second, although we included a wide range of health, demographic and behavioural variables, the CNHS has limited data available regarding other emerging behavioural risk factors, such as consume of processed meat and fizzy drinks, which has limited our ability to take into account their potential confounding effect in the current study or include these variables in the score. Third, lifestyle exposures such as physical activity were self-reported, which have been shown to be prone to bias^(49,50)^. Fourth, the lifestyle score assumed equal weighting for each health metric included. Therefore, future studies could derive item weightings based on strength of associations and validate them using external datasets. Finally, as with all observational studies, causal associations cannot be concluded from this study.

In conclusion, individuals in the less healthy lifestyle category experienced a higher and earlier mortality risk than those with healthier lifestyles. Considering these individuals could die up to almost 11 years early, public health strategies should be implemented to promote adherence to a healthy lifestyle across the Chilean population but especially in those at higher risk, such as women, older adults and people living in urban areas.

As we cannot ignore that the type of lifestyle that people follow matters, we need to encourage healthier behaviours according to the different social, cultural and economic factors that interact in people’s lives. However, one of the enormous problems related to habits is trying to change them during adulthood or when people are already ill. Consequently, private and public sectors, governments and industries, families and individuals and all the stakeholders must work together towards promoting healthier patterns throughout the life cycle.

## Supporting information

Petermann-Rocha et al. supplementary material Petermann-Rocha et al. supplementary material

## Supplementary material

For supplementary material accompanying this paper visit https://doi.org/10.1017/S1368980023002598
