# Supplementary material for: Association between a lifestyle score and all-cause mortality: a prospective analysis of the Chilean National Health Survey 2009–2010
Source: Public Health Nutr. 2023 Dec 6;27(1):e9. doi: 10.1017/S1368980023002598 (PMC10830369; doi:10.1017/S1368980023002598)
Supplement: Petermann-Rocha et al. supplementary material [file S1368980023002598sup001.docx]

**Association between a lifestyle score and all-cause mortality: a prospective analysis of the Chilean National Health Survey 2009-2010**

**Supplementary Table 1. Individual components of the lifestyle score and their association with all-cause mortality**

|  | **HR (95% CI)** | **P-value** |
| --- | --- | --- |
| **Sleep** |  |  |
| Model 3 | 1.23 (0.97; 1.56) | 0.081 |
| Model 4 | 1.21 (0.95; 1.53) | 0.117 |
| **Smoking** |  |  |
| Model 3 | 1.10 (0.86; 1.40) | 0.456 |
| Model 4 | 1.10 (0.86; 1.41) | 0.458 |
| **Physical activity** |  |  |
| Model 3 | 1.60 (1.25; 2.04) | <0.001 |
| Model 4 | 1.51 (1.17; 1.94) | 0.001 |
| **Sedentary behaviour** |  |  |
| Model 3 | 1.72 (1.36; 2.18) | <0.001 |
| Model 4 | 1.58 (1.24; 2.02) | <0.001 |
| **Salt intake** |  |  |
| Model 3 | 1.10 (0.84; 1.44) | 0.483 |
| Model 4 | 1.23 (0.94; 1.61) | 0.139 |
| **Alcohol** |  |  |
| Model 3 | 1.63 (1.07; 2.48) | 0.022 |
| Model 4 | 1.68 (1.10; 2.56) | 0.017 |
| **Fruit and Vegetable** |  |  |
| Model 3 | 0.89 (0.70; 1.15) | 0.385 |
| Model 4 | 0.93 (0.72; 1.20) | 0.582 |

Analyses are presented as HR and their 95% CI. Participants with normal behaviour for each component were used as the reference group. Model 3 was adjusted for age, sex, zone of residency, educational level, diabetes, hypertension, hypertriglyceridemia, and BMI. Model 4 additionally included all the individual components when these were not the exposure (sensitivity analysis).
